# Editor's Message

**DOI:** 10.29173/jchla29613

**Published:** 2022-04-01

**Authors:** Alanna Campbell

It is spring! I am excited to see the signs of spring and say goodbye to the extended cold temperatures of this winter. I know many of you are also. Along with spring comes the April issue of JCHLA/JABSC. We have a great line up this issue including two research papers. The first looking at developing a code of practice for mediated searching. The second looks at mobile device use by medical learners. We also have a number of book and product reviews two of which include a review of the book *Diversity, Equity, and Inclusion in Action: Planning, Leadership, and Programming* as well as a product review of Polyglot Search Translator designed to support your systematic searching.

This April is of particular importance as it concludes the 1-year-trial period of our JCHLA/JABSC Data Sharing Policy. Please note all submissions going forward will be required to follow this policy. For more information view our Data Sharing Policy and our Data Sharing FAQ.

Lastly, don’t forget to register for this year’s CHLA/ABSC Conference 2022. For details you can find an announcement from the Conference Planning Committee in this issue. We’re looking forward to an inspiring program. If you’re presenting a paper or poster, we’d like to invite you to consider submitting it to JCHLA/JABSC – see our Author Guidelines for more on how your work could be featured in our journal.

**Alanna Campbell**

*JCHLA/JABSC Editor-in-Chief*

*Email: editor@chla-absc.ca*

C'est le printemps ! J'ai hâte d’en voir les signes durables et de dire adieu aux températures froides prolongées de cet hiver. Je sais que beaucoup d'entre vous partagent ce sentiment. Avec le printemps vient le numéro d'avril du Journal de l’Association des bibliothèques en santé du Canada / Journal of the Canadian Health Libraries Association (JABSC / JCHLA). Nous proposons un grand choix d’articles pour ce numéro, y compris deux articles de recherche. Le premier porte sur le développement d'un code de pratique pour la recherche médiatisée. Le second porte sur l'utilisation des appareils mobiles par les étudiants en médecine. Nous avons également un certain nombre de critiques de livres et de produits, dont deux présentent une critique du livre *Diversity, Equity, and Inclusion in Action : Planning, Leadership, and Programming* ainsi qu'une revue du produit *Polyglot Search Translator*, pour le soutien aux recherches systématiques.

Ce mois d'avril revêt une importance particulière, car il conclut la période d'essai d'un an de la politique de partage des données du JABSC / JCHLA. Veuillez prendre note que dorénavant toutes les soumissions devront suivre cette politique. Pour plus d'informations, nous vous invitons à consulter la politique de partage des données et la chronique FAQ sur le sujet.

Enfin, n'oubliez pas de vous inscrire au congrès 2022 de l'ABSC / CHLA. Pour plus de détails, vous trouverez une annonce du comité de planification de la conférence, dans ce numéro. Nous attendons avec impatience un programme stimulant. Si vous présentez une communication ou une affiche, nous vous invitons à considérer la soumettre au JABSC / JCHLA - consultez nos directives pour les auteurs afin de savoir comment votre travail pourrait être présenté dans notre journal.

**Alanna Campbell**

*Rédactrice en chef, JABSC / JCHLA*

*Courriel: editor@chla-absc.ca*

